# Supplementary material for: Breastfeeding Duration and High Blood Pressure in Children and Adolescents: Results from a Cross-Sectional Study of Seven Provinces in China
Source: Nutrients. 2022 Jul 30;14(15):3152. doi: 10.3390/nu14153152 (PMC9370455; doi:10.3390/nu14153152)
Supplement: Supplementary file 1 [file nutrients-14-03152-s001.zip › nutrients-1817011-supplementary.pdf]

Supplement file

Supplementary Table S1. Multivariate odds ratios (OR) and 95% confidence intervals (CI) of overweight/obesity by breastfeeding duration.

Breastfeeding duration	Population (%)	Overweight/obesity prevalence (%)	ORs (95%CI)	
			Unadjusted	*Fully adjusted*
**Total population**	57201	5766 (10.08)		
Non-breastfeeding	7086 (12.39)	784 (11.06)	1 (Reference)	1 (Reference)
0-5 months	21953 (38.38)	1998 (9.10)	**0.81 (0.74-0.88)**	0.99 (0.90-1.10)
6-12 months	15273 (26.70)	1369 (8.96)	**0.79 (0.72-0.87)**	**0.80 (0.72-0.88)**
> 12 months	12889 (22.53)	1615 (12.53)	**1.15 (1.05-1.26)**	**1.16 (1.05-1.28)**
**Boys**	29491	2806 (9.51)		
Non-breastfeeding	3652 (12.38)	392 (10.73)	1 (Reference)	1 (Reference)
0-5 months	11735 (39.79)	1021 (8.70)	**0.79 (0.70-0.90)**	0.99 (0.86-1.15)
6-12 months	7585 (25.82)	636 (8.38)	**0.76 (0.67-0.87)**	**0.79 (0.69-0.92)**
> 12 months	6519 (22.11)	757 (11.61)	1.09 (0.96-1.24)	**1.17 (1.01-1.35)**
**Girls**	27710	2960 (10.68)		
Non-breastfeeding	3434 (12.39)	392 (11.42)	1 (Reference)	1 (Reference)
0-5 months	10218 (36.87)	977 (9.56)	**0.82 (0.73-0.93)**	1.00 (0.87-1.15)
6-12 months	7688 (27.74)	733 (9.53)	**0.82 (0.72-0.93)**	**0.80 (0.70-0.92)**
> 12 months	6370 (22.99)	858 (13.47)	**1.21 (1.06-1.37)**	**1.15 (1.00-1.32)**

Fully adjusted Model: adjusted for age, sex, birth weight, single-child status, residence area, maternal age at delivery, parental educational attainment, family history of diseases (hypertension, diabetes, heart disease, cerebrovascular disease and obesity), monthly household income, dietary behaviors (including fruit, vegetable, SSB and meat consumption) and physical activity. Bold values referred to $P<0.05$.

Supplementary Table S2. BP and BP Z scores in breastfeeding duration groups by subgroups.

Subgroups	Average values	Breastfeeding duration groups			
		Non-breastfeeding	0-5 months	6-12 months	> 12 months
**Age**					
7-10 years old					
SBP (mmHg)	101.32±11.27	100.65±11.16	100.75±11.40	100.73±11.15	103.42±11.07
SBP Z-score	-0.32±0.94	-0.38±0.93	-0.37±0.95	-0.37±0.93	-0.15±0.92
DBP (mmHg)	65.07±8.68	64.52±8.35	65.09±9.00	64.42±8.64	66.28±8.31
DBP Z-score	-0.20±1.00	-0.26±0.96	-0.19±1.03	-0.27±0.99	-0.06±0.95
11-14 years old					
SBP (mmHg)	108.13±11.53	107.59±11.57	107.46±11.61	106.28±11.43	111.06±10.95
SBP Z-score	0.25±0.96	0.20±0.96	0.19±0.97	0.09±0.95	0.49±0.91
DBP (mmHg)	67.77±8.65	67.40±8.38	67.59±8.95	66.42±8.47	69.45±8.22
DBP Z-score	0.11±0.99	0.07±0.96	0.09±1.03	-0.04±0.97	0.31±0.94
15-18 years old					
SBP (mmHg)	109.61±11.51	109.28±11.86	109.08±11.43	108.45±11.30	112.62±11.28
SBP Z-score	0.37±0.96	0.34±0.99	0.32±0.95	0.27±0.94	0.62±0.94
DBP (mmHg)	69.18±8.04	68.57±7.98	68.87±8.19	68.46±7.69	71.22±7.72
DBP Z-score	0.28±0.92	0.21±0.92	0.24±0.94	0.19±0.88	0.51±0.89
**Overweight/obesity**					
Yes					
SBP (mmHg)	112.89±12.84	111.81±12.80	112.87±12.95	111.27±12.68	114.81±12.63
SBP Z-score	0.64±1.07	0.55±1.07	0.64±1.08	0.51±1.06	0.80±1.05
DBP (mmHg)	71.18±8.91	70.58±8.54	71.27±9.25	70.13±8.84	72.26±8.59
DBP Z-score	0.51±1.02	0.44±0.98	0.52±1.06	0.38±1.02	0.63±0.99
No					
SBP (mmHg)	104.32±11.59	103.10±11.56	104.31±11.70	102.91±11.35	106.72±11.33
SBP Z-score	-0.07±0.97	-0.17±0.96	-0.07±0.97	-0.19±0.95	0.13±0.94
DBP (mmHg)	66.29±8.54	65.46±8.28	66.47±8.75	65.26±8.42	67.70±8.23
DBP Z-score	-0.06±0.98	-0.15±0.95	-0.04±1.01	-0.17±0.97	0.11±0.95
**Birth weight**					
Low birth weight					
SBP (mmHg)	105.23±12.20	103.88±12.25	105.34±12.26	103.55±11.44	107.42±12.10
SBP Z-score	0.00±1.02	-0.11±1.02	0.01±1.02	-0.14±0.95	0.19±1.01
DBP (mmHg)	67.32±9.24	65.95±8.81	67.58±9.34	65.67±8.64	67.70±8.81
DBP Z-score	0.06±1.06	-0.10±1.01	0.09±1.07	-0.13±0.99	0.11±1.01
Normal birth weight					
SBP (mmHg)	105.02±11.94	103.93±11.95	104.71±11.87	103.51±11.74	107.69±11.81
SBP Z-score	-0.01±0.99	-0.10±1.00	-0.04±0.99	-0.14±0.98	0.21±0.98
DBP (mmHg)	66.57±8.55	65.97±8.43	66.26±8.48	65.66±8.60	68.30±8.38
DBP Z-score	-0.02±0.98	-0.09±0.97	-0.06±0.97	-0.13±0.99	0.17±0.96

High birth weight					
SBP (mmHg)	106.49±11.94	105.67±12.30	106.59±12.20	105.06±11.70	108.26±11.60
SBP Z-score	0.11±0.99	0.04±1.02	0.12±1.02	-0.01±0.97	0.26±0.97
DBP (mmHg)	67.14±8.44	66.66±8.42	67.27±8.46	66.07±8.32	68.36±8.41
DBP Z-score	0.04±0.97	-0.01±0.97	0.06±0.97	-0.08±0.96	0.18±0.97
**Single-child status**					
Yes					
SBP (mmHg)	105.08±12.10	104.10±12.00	105.03±12.14	103.66±11.85	107.81±11.96
SBP Z-score	-0.01±1.01	-0.09±1.00	-0.01±1.01	-0.13±0.99	0.22±1.00
DBP (mmHg)	66.76±8.74	66.02±8.43	66.99±9.00	65.68±8.54	68.18±8.39
DBP Z-score	0.00±1.00	-0.09±0.97	0.02±1.03	-0.13±0.98	0.16±0.96
No					
SBP (mmHg)	105.42±11.77	104.00±12.07	105.37±11.80	103.66±11.45	107.63±11.58
SBP Z-score	0.02±0.98	-0.10±1.00	0.02±0.98	-0.13±0.95	0.20±0.96
DBP (mmHg)	66.85±8.60	66.02±8.56	66.58±8.49	65.75±8.65	68.40±8.44
DBP Z-score	0.01±0.99	-0.09±0.98	-0.02±0.98	-0.12±0.99	0.19±0.97
**Residence area**					
Urban area					
SBP (mmHg)	103.79±11.99	102.94±11.81	103.92±12.14	102.47±11.47	106.13±12.18
SBP Z-score	-0.12±1.00	-0.19±0.98	-0.10±1.01	-0.23±0.95	0.08±1.01
DBP (mmHg)	65.86±8.79	65.13±8.26	66.23±9.09	64.81±8.46	67.14±8.72
DBP Z-score	-0.11±1.01	-0.19±0.95	-0.06±1.04	-0.23±0.97	0.04±1.00
Rural area					
SBP (mmHg)	107.88±11.57	106.68±12.09	108.40±11.26	105.98±11.85	109.36±11.19
SBP Z-score	0.23±0.96	0.13±1.01	0.27±0.94	0.07±0.99	0.35±0.93
DBP (mmHg)	68.57±8.24	68.10±8.58	68.84±8.05	67.42±8.53	69.42±7.93
DBP Z-score	0.21±0.95	0.15±0.99	0.24±0.92	0.07±0.98	0.30±0.91
**Paternal education**					
Primary school or below					
SBP (mmHg)	107.20±11.54	106.44±12.00	107.73±11.88	106.01±11.08	107.90±11.40
SBP Z-score	0.17±0.96	0.11±1.00	0.21±0.99	0.07±0.92	0.23±0.95
DBP (mmHg)	67.76±8.40	67.44±8.44	67.47±8.41	66.85±8.53	68.70±8.20
DBP Z-score	0.11±0.96	0.08±0.97	0.08±0.97	0.01±0.98	0.22±0.94
Secondary or equivalent					
SBP (mmHg)	105.71±11.96	104.69±12.07	105.62±11.99	104.24±11.71	107.85±11.80
SBP Z-score	0.04±1.00	-0.04±1.00	0.04±1.00	-0.08±0.97	0.22±0.98
DBP (mmHg)	67.18±8.77	66.42±8.56	67.39±8.94	66.02±8.73	68.33±8.42
DBP Z-score	0.05±1.01	-0.04±0.98	0.07±1.03	-0.09±1.00	0.18±0.97
Junior college or above					
SBP (mmHg)	102.96±12.01	102.52±11.77	102.02±12.00	102.01±11.67	107.15±12.08
SBP Z-score	-0.18±1.00	-0.22±0.98	-0.26±1.00	-0.26±0.97	0.16±1.01
DBP (mmHg)	65.28±8.39	65.05±8.20	64.57±8.45	64.81±8.18	67.77±8.49

DBP Z-score	-0.17±0.96	-0.20±0.94	-0.25±0.97	-0.23±0.94	0.11±0.98
**Maternal education**					
Primary school or below					
SBP (mmHg)	106.88±11.55	105.59±12.32	107.40±11.61	105.10±11.33	108.29±11.21
SBP Z-score	0.14±0.96	0.03±1.03	0.19±0.97	-0.01±0.94	0.26±0.93
DBP (mmHg)	67.61±8.52	66.53±8.68	67.57±8.54	66.54±8.38	68.81±8.42
DBP Z-score	0.09±0.98	-0.03±1.00	0.09±0.98	-0.03±0.96	0.23±0.97
Secondary or equivalent					
SBP (mmHg)	105.71±11.95	104.74±12.11	105.65±11.99	104.22±11.68	107.82±11.79
SBP Z-score	0.04±1.00	-0.04±1.01	0.04±1.00	-0.08±0.97	0.22±0.98
DBP (mmHg)	67.18±8.75	66.49±8.55	67.40±8.93	66.00±8.71	68.31±8.36
DBP Z-score	0.05±1.01	-0.03±0.98	0.07±1.03	-0.09±1.00	0.18±0.96
Junior college or above					
SBP (mmHg)	102.66±12.02	102.36±11.59	101.45±11.97	101.94±11.73	106.84±12.36
SBP Z-score	-0.21±1.00	-0.23±0.96	-0.31±1.00	-0.27±0.98	0.14±1.03
DBP (mmHg)	65.10±8.41	64.98±8.14	64.23±8.41	64.75±8.25	67.59±8.60
DBP Z-score	-0.19±0.97	-0.21±0.94	-0.29±0.97	-0.23±0.95	0.09±0.99

Supplementary Table S3. Multiple linear regression analysis of breastfeeding duration and BP and BP Z scores in each subgroup, β(95%CI).

Subgroups	Breastfeeding duration	Breastfeeding duration groups			
		Non-breastfeeding	0-5 months	6-12 months	> 12 months
**Age**					
7-10 years old					
SBP (mmHg)	**0.18 (0.14, 0.21)**	1 (Reference)	0.04 (-0.42, 0.50)	0.20 (-0.23, 0.63)	**1.66 (1.20, 2.12)**
SBP Z-score	**0.01 (0.01, 0.02)**	1 (Reference)	0.00 (-0.04, 0.04)	0.02 (-0.02, 0.05)	**0.14 (0.10, 0.18)**
DBP (mmHg)	**0.12 (0.09, 0.15)**	1 (Reference)	-0.20 (-0.56, 0.15)	-0.05 (-0.38, 0.29)	**1.00 (0.65, 1.36)**
DBP Z-score	**0.01 (0.01, 0.02)**	1 (Reference)	-0.02 (-0.06, 0.02)	-0.01 (-0.04, 0.03)	**0.12 (0.07, 0.16)**
11-14 years old					
SBP (mmHg)	**0.39 (0.34, 0.43)**	1 (Reference)	**-0.83 (-1.49, -0.17)**	**-1.27 (-1.89, -0.65)**	**2.16 (1.54, 2.79)**
SBP Z-score	**0.03 (0.03, 0.04)**	1 (Reference)	**-0.07 (-0.12, -0.01)**	**-0.11 (-0.16, -0.05)**	**0.18 (0.13, 0.23)**
DBP (mmHg)	**0.22 (0.19, 0.25)**	1 (Reference)	-0.34 (-0.84, 0.15)	**-0.92 (-1.39, -0.46)**	**1.35 (0.88, 1.83)**
DBP Z-score	**0.03 (0.02, 0.03)**	1 (Reference)	-0.04 (-0.10, 0.02)	**-0.11 (-0.16, -0.05)**	**0.16 (0.10, 0.21)**
15-18 years old					
SBP (mmHg)	**0.29 (0.23, 0.35)**	1 (Reference)	-0.09 (-0.87, 0.68)	-0.59 (-1.36, 0.17)	**2.11 (1.31, 2.90)**
SBP Z-score	**0.02 (0.02, 0.03)**	1 (Reference)	-0.01 (-0.07, 0.06)	-0.05 (-0.11, 0.01)	**0.18 (0.11, 0.24)**
DBP (mmHg)	**0.18 (0.14, 0.22)**	1 (Reference)	-0.18 (-0.72, 0.36)	-0.08 (-0.62, 0.45)	**1.43 (0.88, 1.99)**
DBP Z-score	**0.02 (0.02, 0.03)**	1 (Reference)	-0.02 (-0.08, 0.04)	-0.01 (-0.07, 0.05)	**0.16 (0.10, 0.23)**
**Overweight/obesity**					
Yes					
SBP (mmHg)	**0.29 (0.21, 0.37)**	1 (Reference)	-0.10 (-1.23, 1.02)	-0.68 (-1.77, 0.41)	**1.64 (0.56, 2.73)**
SBP Z-score	**0.02 (0.02, 0.03)**	1 (Reference)	-0.01 (-0.10, 0.08)	-0.06 (-0.15, 0.03)	**0.14 (0.05, 0.23)**
DBP (mmHg)	**0.16 (0.10, 0.22)**	1 (Reference)	-0.25 (-1.06, 0.55)	**-0.82 (-1.61, -0.04)**	0.74 (-0.03, 1.51)
DBP Z-score	**0.02 (0.01, 0.02)**	1 (Reference)	-0.03 (-0.12, 0.06)	**-0.09 (-0.18, 0.00)**	**0.09 (0.00, 0.17)**
No					
SBP (mmHg)	**0.28 (0.25, 0.30)**	1 (Reference)	**-0.44 (-0.79, -0.08)**	**-0.39 (-0.72, -0.05)**	**2.02 (1.67, 2.38)**
SBP Z-score	**0.02 (0.02, 0.03)**	1 (Reference)	**-0.04 (-0.07, -0.01)**	**-0.03 (-0.06, 0.00)**	**0.17 (0.14, 0.20)**

DBP (mmHg)	**0.17 (0.15, 0.19)**	1 (Reference)	**-0.38 (-0.65, -0.11)**	**-0.30 (-0.56, -0.05)**	**1.25 (0.98, 1.51)**
DBP Z-score	**0.02 (0.02, 0.02)**	1 (Reference)	**-0.04 (-0.07, -0.01)**	**-0.03 (-0.06, -0.01)**	**0.14 (0.11, 0.17)**
**Birth weight**					
Low birth weight					
SBP (mmHg)	**0.30 (0.20, 0.39)**	1 (Reference)	-0.45 (-1.59, 0.68)	-0.27 (-1.40, 0.85)	**1.51 (0.28, 2.73)**
SBP Z-score	**0.02 (0.02, 0.03)**	1 (Reference)	-0.04 (-0.13, 0.06)	-0.02 (-0.12, 0.07)	**0.13 (0.02, 0.23)**
DBP (mmHg)	**0.13 (0.05, 0.20)**	1 (Reference)	-0.66 (-1.52, 0.20)	-0.17 (-1.02, 0.69)	0.47 (-0.47, 1.40)
DBP Z-score	**0.01 (0.01, 0.02)**	1 (Reference)	-0.08 (-0.18, 0.02)	-0.02 (-0.12, 0.08)	0.05 (-0.05, 0.16)
Normal birth weight					
SBP (mmHg)	**0.30 (0.27, 0.32)**	1 (Reference)	-0.37 (-0.73, 0.00)	**-0.42 (-0.76, -0.07)**	**2.13 (1.77, 2.50)**
SBP Z-score	**0.02 (0.02, 0.03)**	1 (Reference)	-0.03 (-0.06, 0.00)	**-0.03 (-0.06, -0.01)**	**0.18 (0.15, 0.21)**
DBP (mmHg)	**0.17 (0.15, 0.19)**	1 (Reference)	**-0.38 (-0.65, -0.10)**	**-0.35 (-0.61, -0.09)**	**1.26 (0.99, 1.53)**
DBP Z-score	**0.02 (0.02, 0.02)**	1 (Reference)	**-0.04 (-0.07, -0.01)**	**-0.04 (-0.07, -0.01)**	**0.14 (0.11, 0.18)**
High birth weight					
SBP (mmHg)	**0.17 (0.10, 0.25)**	1 (Reference)	-0.97 (-2.07, 0.13)	-0.80 (-1.84, 0.24)	0.79 (-0.26, 1.85)
SBP Z-score	**0.01 (0.01, 0.02)**	1 (Reference)	-0.08 (-0.17, 0.01)	-0.07 (-0.15, 0.02)	0.07 (-0.02, 0.15)
DBP (mmHg)	**0.12 (0.06, 0.17)**	1 (Reference)	-0.40 (-1.23, 0.43)	-0.75 (-1.53, 0.03)	0.60 (-0.20, 1.39)
DBP Z-score	**0.01 (0.01, 0.02)**	1 (Reference)	-0.05 (-0.14, 0.05)	-0.09 (-0.18, 0.00)	0.07 (-0.02, 0.16)
**Single-child status**					
Yes					
SBP (mmHg)	**0.29 (0.26, 0.32)**	1 (Reference)	**-0.61 (-1.01, -0.21)**	**-0.42 (-0.80, -0.04)**	**2.10 (1.69, 2.50)**
SBP Z-score	**0.02 (0.02, 0.03)**	1 (Reference)	**-0.05 (-0.08, -0.02)**	**-0.03 (-0.07, 0.00)**	**0.17 (0.14, 0.21)**
DBP (mmHg)	**0.17 (0.15, 0.19)**	1 (Reference)	**-0.48 (-0.78, -0.18)**	**-0.37 (-0.66, -0.09)**	**1.18 (0.88, 1.49)**
DBP Z-score	**0.02 (0.02, 0.02)**	1 (Reference)	**-0.05 (-0.09, -0.02)**	**-0.04 (-0.08, -0.01)**	**0.14 (0.10, 0.17)**
No					
SBP (mmHg)	**0.24 (0.20, 0.28)**	1 (Reference)	0.07 (-0.57, 0.71)	-0.35 (-0.95, 0.25)	**1.75 (1.15, 2.36)**
SBP Z-score	**0.02 (0.02, 0.02)**	1 (Reference)	0.01 (-0.05, 0.06)	-0.03 (-0.08, 0.02)	**0.15 (0.10, 0.20)**
DBP (mmHg)	**0.16 (0.12, 0.19)**	1 (Reference)	-0.12 (-0.61, 0.36)	-0.29 (-0.75, 0.17)	**1.19 (0.73, 1.65)**
DBP Z-score	**0.02 (0.01, 0.02)**	1 (Reference)	-0.01 (-0.07, 0.04)	-0.03 (-0.09, 0.02)	**0.14 (0.08, 0.19)**

**Residence area**					
Urban area					
SBP (mmHg)	**0.31 (0.28, 0.35)**	1 (Reference)	**-0.92 (-1.34, -0.50)**	-0.32 (-0.72, 0.07)	**2.32 (1.89, 2.75)**
SBP Z-score	**0.03 (0.02, 0.03)**	1 (Reference)	**-0.08 (-0.11, -0.04)**	-0.03 (-0.06, 0.01)	**0.19 (0.16, 0.23)**
DBP (mmHg)	**0.19 (0.16, 0.21)**	1 (Reference)	**-0.67 (-0.99, -0.36)**	-0.21 (-0.51, 0.09)	**1.49 (1.17, 1.81)**
DBP Z-score	**0.02 (0.02, 0.02)**	1 (Reference)	**-0.08 (-0.11, -0.04)**	-0.02 (-0.06, 0.01)	**0.17 (0.13, 0.21)**
Rural area					
SBP (mmHg)	**0.23 (0.20, 0.27)**	1 (Reference)	**0.58 (0.00, 1.16)**	-0.40 (-0.95, 0.16)	**1.57 (1.01, 2.12)**
SBP Z-score	**0.02 (0.02, 0.02)**	1 (Reference)	**0.05 (0.00, 0.10)**	-0.03 (-0.08, 0.01)	**0.13 (0.08, 0.18)**
DBP (mmHg)	**0.14 (0.12, 0.17)**	1 (Reference)	0.22 (-0.22, 0.65)	**-0.54 (-0.96, -0.12)**	**0.79 (0.37, 1.20)**
DBP Z-score	**0.02 (0.01, 0.02)**	1 (Reference)	0.02 (-0.03, 0.08)	**-0.06 (-0.11, -0.01)**	**0.09 (0.04, 0.14)**
**Paternal education**					
Primary school or below					
SBP (mmHg)	**0.16 (0.08, 0.25)**	1 (Reference)	0.28 (-1.14, 1.70)	-0.67 (-2.05, 0.71)	0.74 (-0.58, 2.06)
SBP Z-score	**0.01 (0.01, 0.02)**	1 (Reference)	0.02 (-0.10, 0.14)	-0.06 (-0.17, 0.06)	0.06 (-0.05, 0.17)
DBP (mmHg)	**0.15 (0.08, 0.21)**	1 (Reference)	-0.31 (-1.39, 0.77)	-0.57 (-1.62, 0.48)	0.89 (-0.11, 1.90)
DBP Z-score	**0.02 (0.01, 0.02)**	1 (Reference)	-0.04 (-0.16, 0.09)	-0.07 (-0.19, 0.06)	0.10 (-0.01, 0.22)
Secondary or equivalent					
SBP (mmHg)	**0.26 (0.23, 0.29)**	1 (Reference)	-0.20 (-0.64, 0.24)	**-0.43 (-0.84, -0.02)**	**1.78 (1.36, 2.20)**
SBP Z-score	**0.02 (0.02, 0.02)**	1 (Reference)	-0.02 (-0.05, 0.02)	**-0.04 (-0.07, 0.00)**	**0.15 (0.11, 0.18)**
DBP (mmHg)	**0.15 (0.13, 0.17)**	1 (Reference)	-0.21 (-0.54, 0.12)	**-0.44 (-0.76, -0.13)**	**0.98 (0.66, 1.30)**
DBP Z-score	**0.02 (0.01, 0.02)**	1 (Reference)	-0.02 (-0.06, 0.01)	**-0.05 (-0.09, -0.01)**	**0.11 (0.08, 0.15)**
Junior college or above					
SBP (mmHg)	**0.34 (0.29, 0.39)**	1 (Reference)	**-1.00 (-1.57, -0.42)**	-0.38 (-0.93, 0.17)	**3.00 (2.36, 3.65)**
SBP Z-score	**0.03 (0.02, 0.03)**	1 (Reference)	**-0.08 (-0.13, -0.04)**	-0.03 (-0.08, 0.01)	**0.25 (0.20, 0.30)**
DBP (mmHg)	**0.21 (0.17, 0.25)**	1 (Reference)	**-0.73 (-1.16, -0.30)**	-0.18 (-0.59, 0.22)	**1.88 (1.40, 2.36)**
DBP Z-score	**0.02 (0.02, 0.03)**	1 (Reference)	**-0.08 (-0.13, -0.03)**	-0.02 (-0.07, 0.03)	**0.22 (0.16, 0.27)**
**Maternal education**					
Primary school or below					

SBP (mmHg)	**0.21 (0.13, 0.28)**	1 (Reference)	0.70 (-0.51, 1.90)	-0.94 (-2.10, 0.23)	**1.36 (0.24, 2.49)**
SBP Z-score	**0.02 (0.01, 0.02)**	1 (Reference)	0.06 (-0.04, 0.16)	-0.08 (-0.18, 0.02)	**0.11 (0.02, 0.21)**
DBP (mmHg)	**0.17 (0.11, 0.22)**	1 (Reference)	0.47 (-0.47, 1.40)	-0.26 (-1.16, 0.64)	**1.37 (0.50, 2.24)**
DBP Z-score	**0.02 (0.01, 0.03)**	1 (Reference)	0.05 (-0.05, 0.16)	-0.03 (-0.13, 0.07)	**0.16 (0.06, 0.26)**
Secondary or equivalent					
SBP (mmHg)	**0.25 (0.22, 0.28)**	1 (Reference)	-0.20 (-0.64, 0.24)	**-0.42 (-0.84, -0.01)**	**1.74 (1.32, 2.16)**
SBP Z-score	**0.02 (0.02, 0.02)**	1 (Reference)	-0.02 (-0.05, 0.02)	**-0.04 (-0.07, 0.00)**	**0.14 (0.11, 0.18)**
DBP (mmHg)	**0.15 (0.12, 0.17)**	1 (Reference)	-0.25 (-0.58, 0.08)	**-0.47 (-0.78, -0.16)**	**0.94 (0.62, 1.26)**
DBP Z-score	**0.02 (0.01, 0.02)**	1 (Reference)	-0.03 (-0.07, 0.01)	**-0.05 (-0.09, -0.02)**	**0.11 (0.07, 0.14)**
Junior college or above					
SBP (mmHg)	**0.35 (0.30, 0.40)**	1 (Reference)	**-1.19 (-1.78, -0.60)**	-0.31 (-0.88, 0.25)	**2.95 (2.27, 3.62)**
SBP Z-score	**0.03 (0.02, 0.03)**	1 (Reference)	**-0.10 (-0.15, -0.05)**	-0.03 (-0.07, 0.02)	**0.25 (0.19, 0.30)**
DBP (mmHg)	**0.21 (0.17, 0.25)**	1 (Reference)	**-0.86 (-1.31, -0.42)**	-0.19 (-0.61, 0.23)	**1.86 (1.36, 2.36)**
DBP Z-score	**0.02 (0.02, 0.03)**	1 (Reference)	**-0.10 (-0.15, -0.05)**	-0.02 (-0.07, 0.03)	**0.21 (0.16, 0.27)**

Fully adjusted Model: adjusted for age, sex, birth weight, single-child status, overweight/obesity status, residence area, maternal age at delivery, parental educational attainment, family history of diseases (hypertension, diabetes, heart disease, cerebrovascular disease and obesity), monthly household income, dietary behaviors (including fruit, vegetable, SSB and meat consumption) and physical activity. Bold values referred to $P<0.05$.

Supplementary Table S4. Prevalence of HBP by breastfeeding duration in different subgroups.

Subgroups	Population (%)	HBP Prevalence (%)			
		Non-breastfeeding	0-5 months	6-12 months	> 12 months
**Age**					
7-10 years old	27541 (48.15)	449 (11.52)	1096 (11.77)	909 (11.02)	830 (13.65)
11-14 years old	16897 (29.54)	310 (15.86)	1019 (16.63)	564 (13.06)	978 (21.75)
15-18 years old	12763 (22.31)	227 (18.37)	1244 (19.10)	427 (15.79)	656 (28.39)
**Overweight/obesity**					
Yes	5766 (10.08)	238 (30.36)	690 (34.53)	385 (28.12)	645 (39.94)
No	51435 (89.92)	748 (11.87)	2669 (13.38)	1515 (10.90)	1819 (16.13)
**Birth weight**					
Low birth weight	12672 (22.15)	107 (14.78)	1750 (17.58)	136 (11.55)	147 (18.04)
Normal birth weight	39961 (69.86)	791 (13.70)	1426 (13.11)	1568 (12.41)	2045 (19.14)
High birth weight	4568 (7.99)	88 (14.92)	183 (16.30)	196 (13.38)	272 (19.57)
**Single-child status**					
Yes	40633 (71.04)	704 (13.84)	2768 (15.59)	1252 (12.17)	1450 (19.32)
No	16568 (28.96)	282 (14.09)	591 (14.09)	648 (12.99)	1014 (18.83)
**Residence area**					
Urban area	37739 (65.98)	602 (12.16)	2321 (14.30)	1074 (10.66)	1133 (17.47)
Rural area	19462 (34.02)	384 (17.98)	1038 (18.12)	826 (15.90)	1331 (20.78)
**Paternal education**					
Primary school or below	3539 (6.19)	62 (15.54)	151 (16.54)	131 (14.36)	242 (18.40)
Secondary or equivalent	40716 (71.18)	650 (15.01)	2797 (16.19)	1301 (13.42)	1803 (19.16)
Junior college or above	12946 (22.63)	274 (11.63)	411 (10.93)	468 (10.03)	419 (19.36)
**Maternal education**					
Primary school or below	4829 (8.44)	79 (14.16)	212 (17.00)	168 (12.80)	348 (20.33)
Secondary or equivalent	40579 (70.94)	670 (15.50)	2796 (16.21)	1322 (13.60)	1758 (18.94)
Junior college or above	11793 (20.62)	237 (10.75)	351 (10.16)	410 (9.67)	358 (18.91)
